# Minimally invasive tricuspid valve surgery and concomitant MAZE procedure with closure of LA appendage through an ASD

**DOI:** 10.1002/ccr3.3508

**Published:** 2020-11-11

**Authors:** Guohao Chang, Giap Swee Kang, Christos George Alexiou, Theodoros Kofidis

**Affiliations:** ^1^ Department of Cardiac, Thoracic and Vascular Surgery National University Heart Centre Singapore Singapore; ^2^ Department of Cardiac Surgery Interbalkan Medical Centre Thessaloniki Greece; ^3^ Yong Loo Lin School of Medicine National University of Singapore Singapore Singapore

**Keywords:** atrial septal defect, MAZE procedure, minimally invasive

## Abstract

Utilization of the ASD as a gateway to reach the left heart in tricuspid valve surgery may facilitate the use of a mini right thoracotomy and single atriotomy approach, avoiding the need for bi‐atrial incisions and/or median sternotomy.

## Graphical abstract

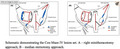

## 1 CASE HISTORY

A 70‐year‐old patient with chronic atrial fibrillation, a large atrial septal defect (ASD), and severe tricuspid regurgitation of rheumatic etiology was admitted to our unit for elective surgery, through a mini right thoracotomy (RT). In this patient, the ASD provided us with a “natural” transseptal route to access the left atrium in order to obliterate the LA appendage and perform a maze procedure, before closing the ASD and replacing the fibrotic and calcified tricuspid valve (TV). Utilization of the ASD as a gateway to reach the left heart in such cases may facilitate the use of a mini RT and single atriotomy approach, avoiding the need for bi‐atrial incisions and/or median sternotomy.

**FIGURE 1:**
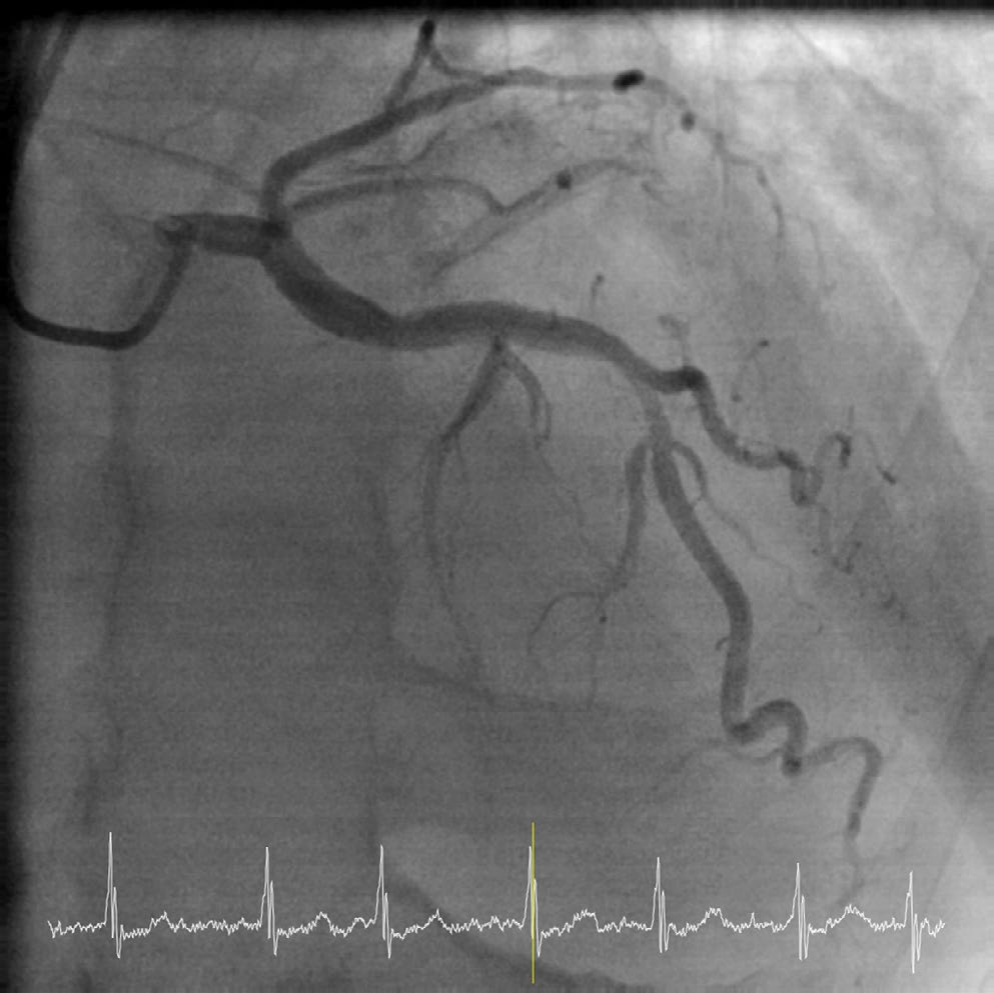
Preoperative Coronary Angiogram: Single Vessel Coronary artery disease showing 50% stenosis of mid‐left anterior descending (LAD) artery

**FIGURE 2:**
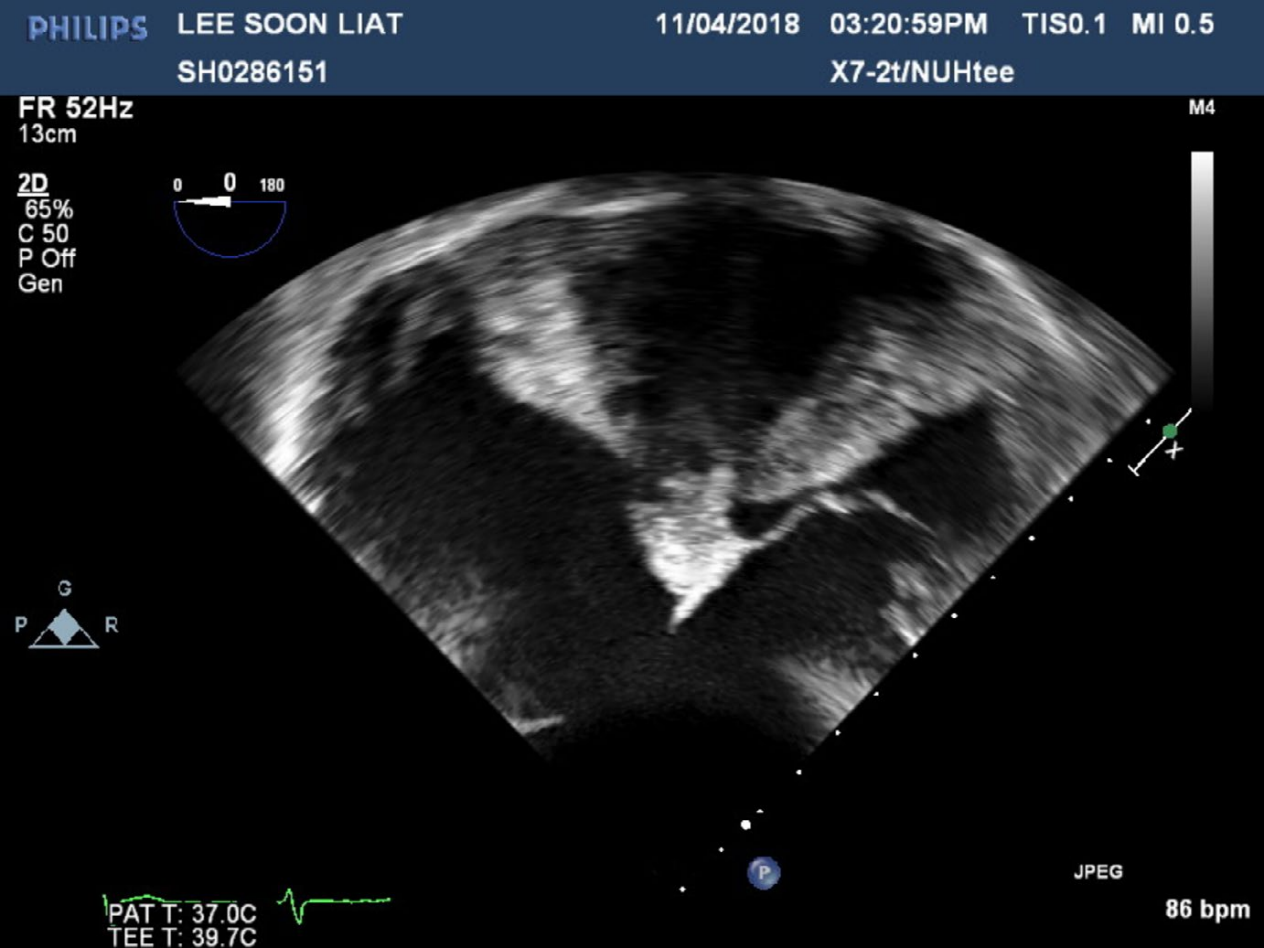
Preoperative Transesophageal Echocardiogram showing ASD

**FIGURE 3:**
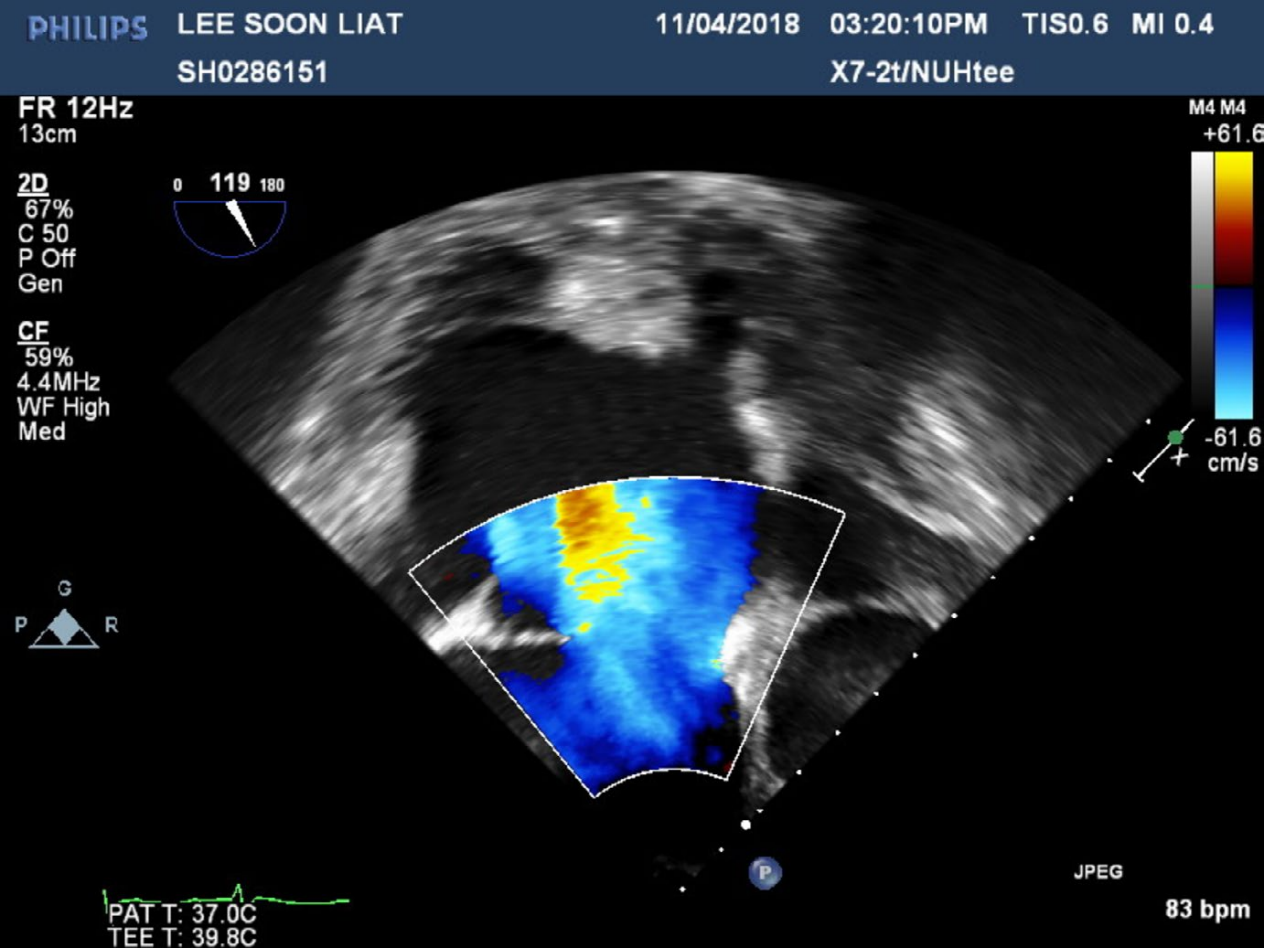
Preoperative Transesophageal Echocardiogram showing color flow across ASD

**FIGURE 4:**
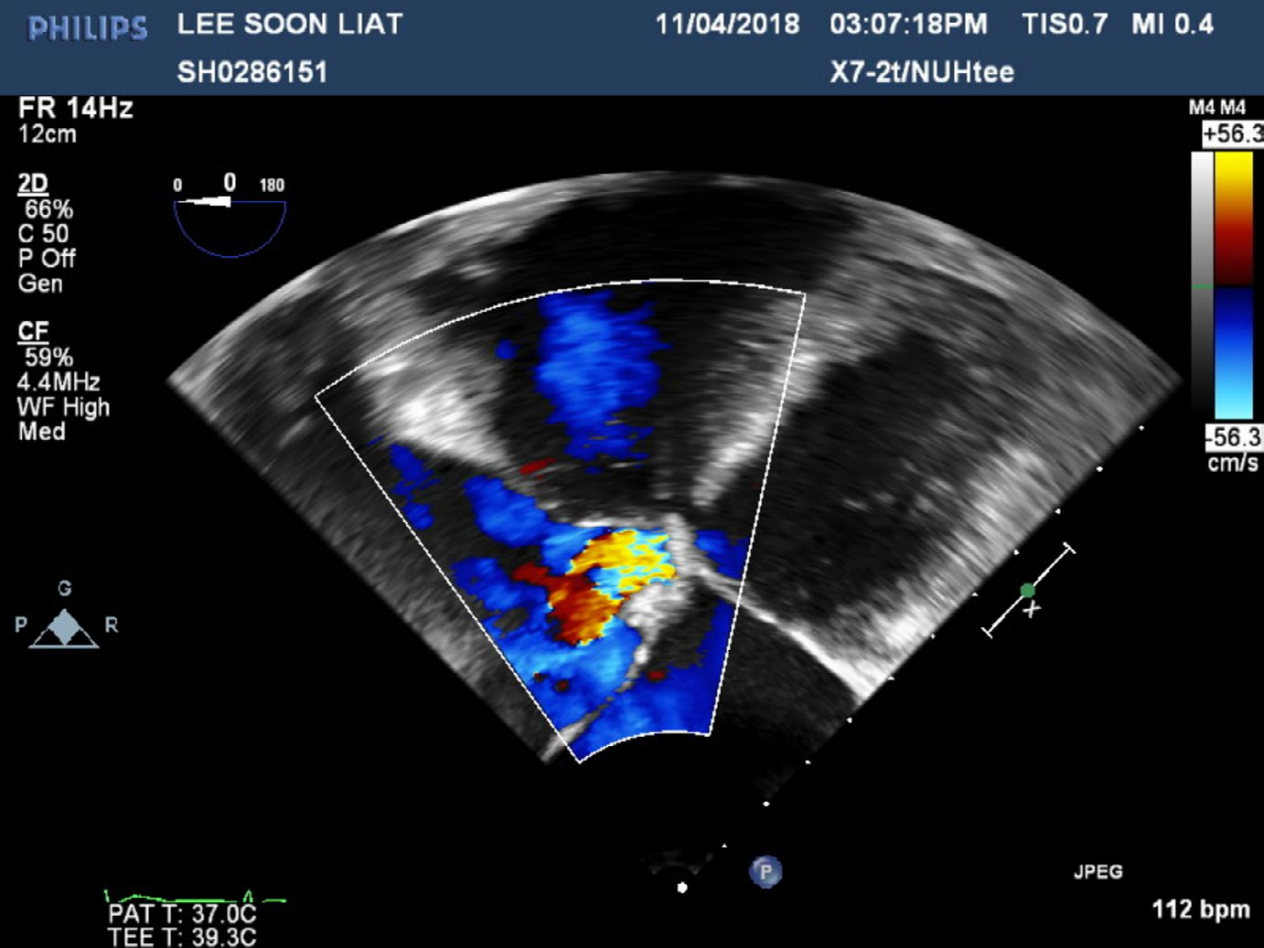
Preoperative Transesophageal Echocardiogram showing TR

A 70‐year‐old Chinese gentleman with atrial septal defect (ASD), tricuspid regurgitation (TR), and atrial fibrillation (AF) of 1.5 years duration, presented with symptoms of dyspnea (NYHA II‐III) and fatigue on minimal exertion despite optimal medical therapy. Past medical history included hypertension and chronic kidney disease. Examination findings revealed bipedal edema, hepatomegaly, and fine basal crepitations bilaterally. Chest X‐ray showed cardiomegaly and pronounced pulmonary vasculature. Preoperative coronary angiogram was done showing 50% stenosis in the mid‐left anterior descending artery (Figure 1). Trans‐esophageal echocardiogram (TOE) demonstrated a 3 cm large ASD with left to right shunt (Qp:Qs 2:3) as seen on Figures 2 and 3, severely dilated and moderately impaired right ventricle (RV), severe tricuspid regurgitation (Figure 4), pulmonary artery systolic pressure 54 mm Hg, mild mitral regurgitation, dilated left atrium (LA), and preserved left ventricular (LV) ejection fraction (EF 55%).

**FIGURE 5:**
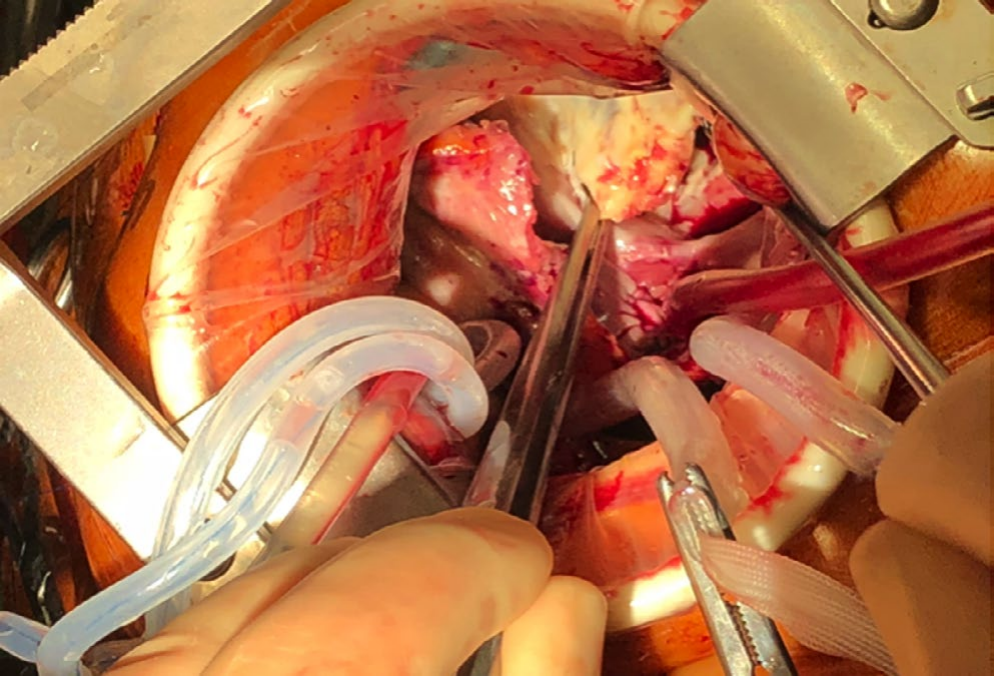
Intraoperative photo: Thick‐walled right atrium and rheumatic changes on the anterior and posterior leaflets of the tricuspid valve

He underwent minimally invasive MAZE, left atrial appendage (LAA) obliteration, patch closure of ASD, and bioprosthetic tricuspid valve replacement (TVR). Access was via a 5 cm long right thoracotomy in the 4th intercostal space. Cardiopulmonary bypass (CPB) was established via cannulation of the right common femoral vein (24 Fr venous cannula—Edwards Lifesciences), the right internal jugular vein (18 Fr Optisite cannula—Edwards Lifesciences), and the right common femoral artery (22 Fr EOPA cannula—Medtronic Inc). After initiation of CPB (nasal temperature of 34 degrees centigrade), venae cavae were snared, Chitwood aortic cross clamp applied, and 1 L of Del Nido cardioplegia was given antegradely down the aortic root. Operative field was flooded with carbon dioxide. The right, dilated and thick‐walled, atrium was opened revealing a large ASD with deficient superior rim. Tricuspid valve annulus was normal with thickened, fused, and calcified anterior and posterior TV leaflets (Figure 5).

**FIGURE 6:**
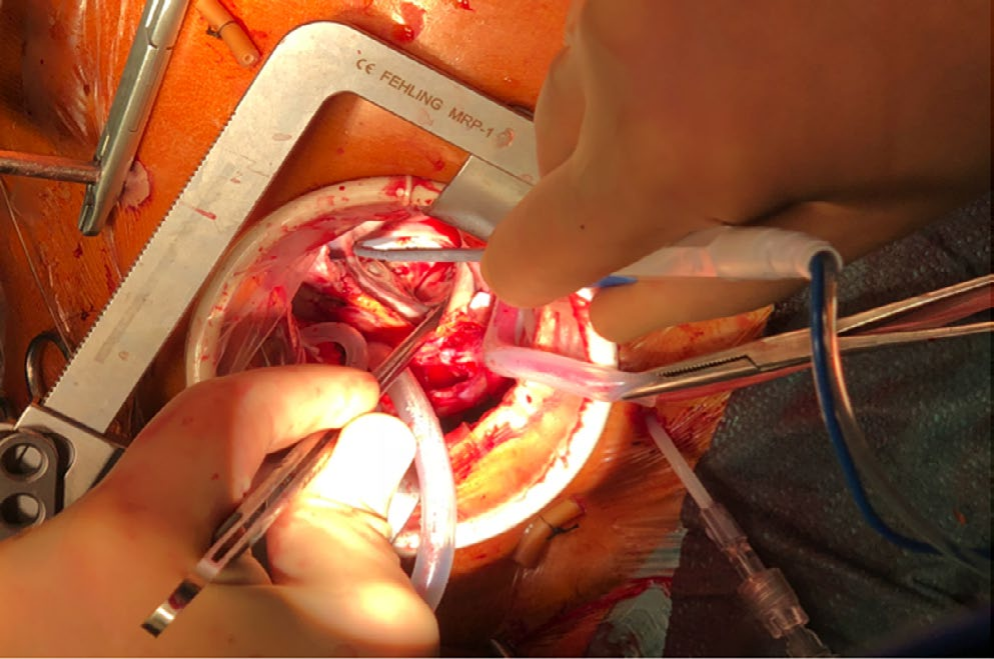
Intraoperative photo: Left atrial maze lesion sets were performed via the atrial septal defect using unipolar radiofrequency ablation

**FIGURE 7:**
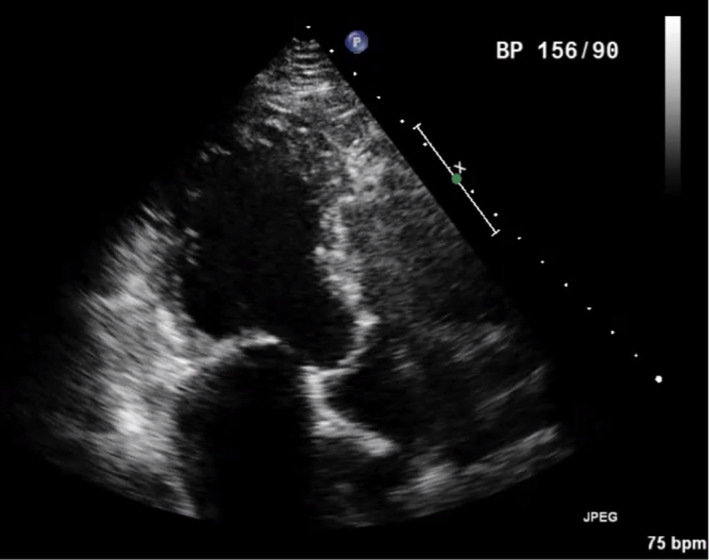
Postoperative Transesophageal Echocardiogram showing absence of LAA after ligation

Right pulmonary vein isolation using bipolar radiofrequency ablation device (Cardioblate^®^, Medtronic Inc) was performed. LA MAZE lesion sets were carried out through the large ASD using unipolar radiofrequency ablation device (Cardioblate^®^, Medtronic Inc) (Figure 6). LAA was obliterated with double‐layered continuous 4/0 polypropylene suture. ASD was next closed with bovine pericardial patch using continuous single‐layered 4/0 polypropylene suture with a 5 × 5 mm cruciate fenestration made centrally. In view of severe rheumatic changes, fibrotic, calcified anterior and posterior TV leaflets were excised. A 33 mm St Jude Epic (St Jude Medical) porcine bioprosthesis was implanted with interrupted pledgetted 2/0 Ethibond (Ethicon) mattressed sutures. The patient was separated from CPB uneventfully with minimal inotropes in sinus rhythm. CPB time was 175 minutes, and aortic crossclamp time was 106 minutes. Post‐repair TOE confirmed complete obliteration of the LAA (Figure 7), presence of an intact pericardial patch with central fenestration, and a well‐seated, normally functioning tricuspid bioprosthesis without paravalvular leak.

**FIGURE 8:**
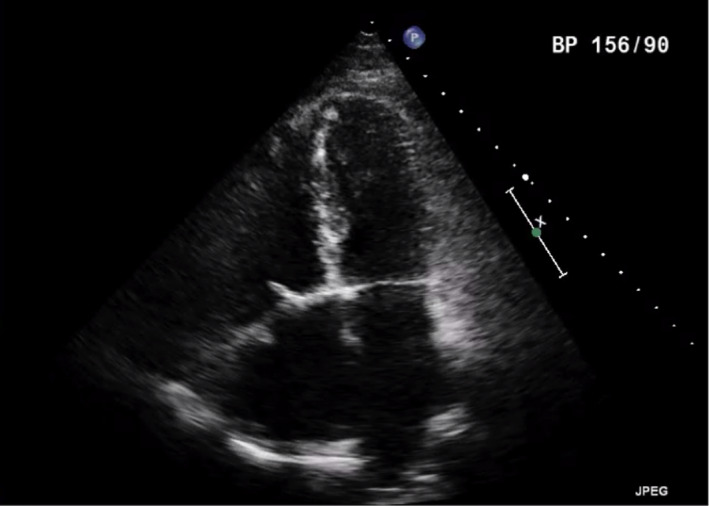
Postoperative Transthoracic Echocardiogram showing TVR

Postoperatively, the patient remained in sinus rhythm and made an uneventful recovery; he stayed in ICU for 24 hours and was discharged home 4 days after his operation. Warfarin was commenced for him with a target international normalized ratio (INR) of 2‐2.5. 3 months later, warfarin was changed to rivaroxaban. At 1‐year follow‐up, he was doing well‐being asymptomatic (NYHA Class I) in sinus rhythm. Transthoracic echocardiogram (TTE) showed no residual shunt, a well‐functioning tricuspid valve (Figure 8) with mean gradient of 3.9 mm Hg, mild RV impairment with moderate RV dilatation and good LV function.

## 2 DISCUSSION

Minimally invasive ASD closure and TVR through a right mini‐thoracotomy in the 4th intercostal space is a widely accepted surgical option.^1^, ^2^ We report the use of the presence of a large ASD to access the left‐sided heart structures in order to close the LAA and to perform a MAZE radiofrequency ablation procedure.

Although utilization of this access route may appear (and it probably is) obvious, to the best of our knowledge, performance of this set of procedures through a right mini‐thoracotomy and single atriotomy has not been previously reported in the literature.

Cleary, a median sternotomy in this case would have been a simpler approach that would enable the surgeon to readily access the left atrium via the “natural” transeptal route provided by the ASD. Another option for those practicing Minimally Invasive Cardiac Surgery (MICS) MICS would have been to perform a right mini‐thoracotomy and to accomplish the described sets of procedures through separate right and left atriotomies. In this patient, we elected to use the large ASD as a gate to reach and close the LAA and to perform left atrial “box” lesions. In this way, it was possible to simplify the operation avoiding either a bi‐atrial MICS or a median sternotomy approach.

The left atrium was adequately visualized through the large ASD, allowing for a straightforward suturing and obliteration of the LAA from within. This could have been also done externally using the AtriClip^3^; however, the exposure afforded through a mini‐thoracotomy alone might not have guaranteed a safe and effective application of the AtriClip down to the base of the LAA.

**FIGURE 9:**
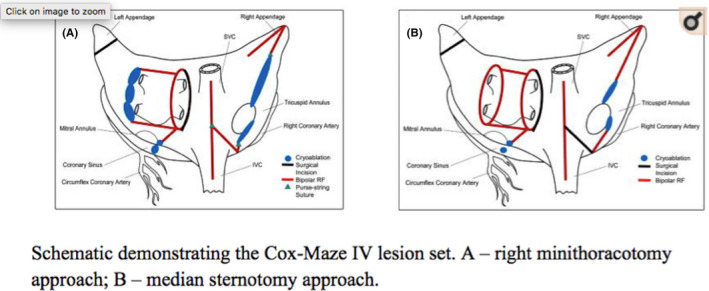
Schematic demonstrating the Cox‐Maze IV lesion set. A, right mini‐thoracotomy approach; (B) median sternotomy approach^4^

The left atrial “box” lesion was comfortably created (Figure 9)^4^ using a linear cryoablation probe behind the left pulmonary veins, across the lateral ridge, connecting the floor and roof lesions. The same linear probe was used to make an endocardial lesion to the mitral annulus.

Management of ASD in adult patients remains controversial; however, appropriately selected patients can derive symptomatic and prognostic benefit regardless of their age at the time of the diagnosis and repair of ASD.^5^ We believe in individualization of treatment following discussion in a multidisciplinary cardiology‐cardiac surgical meeting. The decision was made to fashion a fenestration in the patch repair of the ASD so as to avoid excessive overload to the right ventricle in the immediate postoperative period.

Repair of tricuspid valve is superior to tricuspid valve replacement ^6^ and our priority is to repair rather than replace intracardiac valves if feasible. However, on this occasion, the presence of calcification and fibrosis of anterior and posterior leaflets in this patient dictated the need for replacement of the native tricuspid valve with a bioprosthesis.

## 3 CONCLUSIONS

In summary, in patients undergoing closure of an ASD and TV surgery, concomitant LAA closure and a MAZE procedure can be performed through the ASD via a right mini‐thoracotomy single atriotomy approach avoiding thus the need for bi‐atrial incisions and/or median sternotomy. This procedure would pose no technical challenge to the MICS surgeons; hence, the intention of this report is to describe it and kindly bring it to the attention of the MICS community.

## CONFLICT OF INTEREST

The authors reported no conflict of interest.

## AUTHOR CONTRIBUTIONS

TK: contributed to concept; GC: contributed to data collection and drafting; CA, GC, and GSK: contributed to revision; TK: contributed to approval.

## ETHICAL APPROVAL

Case report is exempted from ethics review approval.
